# Stethoscope disinfection is rarely done in Ethiopia: What are the associated factors?

**DOI:** 10.1371/journal.pone.0208365

**Published:** 2019-06-27

**Authors:** Biniyam Sahiledengle

**Affiliations:** Department of Public Health, Madda Walabu University Goba Referral Hospital, Bale-Goba, Ethiopia; Public Health England, UNITED KINGDOM

## Abstract

**Introduction:**

The stethoscope, which is non-critical medical devices and a symbol of healthcare, is likely to be contaminated by pathogenic microorganisms and can play a contributory role in the transmission of hospital-acquired infection. And regular cleaning of the diaphragm of the stethoscope with a suitable disinfectant is decisive. However, in the resource-constrained setting like many healthcare facilities in Ethiopia healthcare provider’s stethoscope disinfection practice and its associated factors have not been well studied so far. Therefore, this study sought to determine stethoscope disinfection practice and associated factors among the healthcare providers in Addis Ababa, Ethiopia.

**Methods:**

A facility-based cross-sectional study was carried out between April and May 2016. For this study, 576 healthcare providers (physicians, health officers, nurses, midwives, and anesthesiologist) were included from 21 healthcare facilities in Addis Ababa. A pre-tested structured questionnaire was used for data collection. Descriptive statistics were computed. Bivariate and multivariable logistic regression analyses were used to identify factors that were significantly associated with stethoscope disinfection after every use.

**Results:**

A total of 546 healthcare providers participated in this study, for a response rate of 94.7%. Two-fifths, 39.7% (95%CI: 35.9, 44.0%) of healthcare providers disinfecting their stethoscope after every use. And a significant number of participants 34.6% (95%CI: 30.8, 38.5%) never disinfect their stethoscope. Three out of four (76.0%) healthcare providers believe that stethoscope contamination can contribute to the transmission of infections. Safe infection prevention practice (AOR = 3.79, 95%CI: 2.45–5.84), awareness on infection prevention guideline (AOR = 1.93; 95%CI: 1.31, 2.82), and favorable attitude towards infection prevention (AOR = 1.73, 95%CI: 1.02, 2.93) were significantly associated with stethoscope disinfection after every use. The study also found that the odds of stethoscope disinfection were likely to be reduced by 79% among physicians than nurses (AOR = 0.21; 95%CI: 0.09, 0.49).

**Conclusions:**

Only a small proportion of healthcare providers disinfect their stethoscopes after every use. Factors such as safe infection prevention practice, awareness on infection prevention guidelines, and favorable attitude towards infection prevention were the independent predictors of stethoscopes disinfection after every use. Hence, promotion of stethoscope hygiene along with an educational program to enhance disinfection compliance in healthcare facilities may have a positive effect.

## Introduction

Healthcare-associated infections (HCAIs) continues to be one of the most important public health problems in many countries throughout the world [1,2], and remain common in both developed and developing countries [3]. Further, HCAIs result in protracted hospital stays and increased the substantial cost for the healthcare system [4, 5]. It is known long ago HCAIs are caused by viral, bacterial, and fungal pathogens. And many healthcare-associated bacterial pathogens may well survive or persist on different surfaces for months and can thereby be a continuous source of transmission if no regular preventive surface disinfection is performed [6,7]. Investigators of a systematic review also reported highly virulent microorganisms, particularly those known to cause nosocomial infections in admitted patients, such as *Enterococcus* species, *Methicillin-resistant Staphylococcus aureus (MRSA)*, *Escherichia coli*, *Klebsiella* species, *Pseudomonas aeruginosa*, and *Acinetobacter* species, are capable of surviving for several days on hospital surfaces [7]. Consequently, medical equipment surfaces, such as stethoscopes can easily be contaminated with these infectious agents and contribute to the spread of HCAIs [8,9,10,11,12], and may possibly cause outbreaks of hospital-acquired infections [13].

In this regard, the stethoscope, which is universally used as a medical device by healthcare workers, is more likely to be contaminated by microorganisms, if it is not disinfected and may transmit pathogens from one patient to another [14–19]. For this reason, regular cleaning with a suitable disinfectant is decisive and part of a multi-barrier strategy to prevent HCAIs; since failure to properly disinfect or sterilize equipment carries not the only risk associated with breach of host barriers but also risk for person-to-person transmission and transmission of environmental pathogens (e.g., *Pseudomonas aeruginosa*) [14, 20].

There is strong scientific evidence documenting the various pathogens found colonizing stethoscope surfaces [14,15,21,22]. Several studies have demonstrated that stethoscope membranes harbor bacteria, including resistant microorganisms such as *Methicillin-resistant Staphylococcus aureus (MRSA)*, *Vancomycin-resistant Enterococci (VRE)* and *Clostridium difficile* [14,23,24,25]. It was also reported that highly resistant bacteria, MRSA can potentially survive up to 9 days on stethoscopes [26].

Effective disinfection of stethoscopes with isopropyl alcohol eliminates up to 99% of bacteria [22]. However, in the resource-constrained setting like many healthcare facilities in Ethiopia, healthcare workers infection prevention compliance [27,28,29], and instrument disinfection practice was poor [30]. In addition, a high prevalence of HCAIs was noted in hospitalized patients across the country; even if it is difficult to say that HCAI in Ethiopia is high as a result of stethoscope disinfection [31,32,33]. Yet, the available literature evidenced there is significant bacterial contaminations of stethoscopes in the country [34,35,36]. For example, a study by Shiferaw et al [34] reported that of the 176 stethoscopes examined 85.8% were contaminated and of 256 bacteria isolates, 52% were potential pathogens like *S*.*aureus*, *Klebsiella spp*., *Citrobacter spp*., *P*.*aeruginosa*, and *E*.*coli*. Although this evidence is available, there has still been a lack of studies about stethoscope disinfection in Ethiopia to overcome the problem. All the previously conducted related studies are case studies in one or similar type of hospital [34,35,36]. As well as none of the previously conducted studied include health centers in their assessment, where the vast majority of the population seeks healthcare service. According to Ethiopian health tier system health centers where the primary care level establishments providing health care services; such as family planning, maternal and child health service, delivery service, emergency and minor surgery service, pharmacy, laboratory, tuberculosis treatment service, and outpatient service. To the best of my knowledge, this study is the most extensive study, investigating stethoscope disinfection practice in both hospitals and health centers. In order to improve healthcare provider’s appropriate disinfection practice and to develop successful infection prevention programs in-depth understanding of the issues is essential. Therefore, this study aimed to assess the practice of stethoscope disinfection among healthcare providers working in healthcare facilities in Addis Ababa, Ethiopia. Furthermore, the study aimed to identify factors associated with stethoscope disinfection. The findings of the study will be used as an input for policy makers, programmers and healthcare workers to improve quality services and to prioritize interventions by decision makers to overcome the problem.

## Materials and methods

### Study area and design

A facility based cross-sectional study was conducted from April 11 to May 20, 2016, in healthcare facilities of Addis Ababa (the capital city of Ethiopia). In this study, a total of 21 healthcare facilities (3 hospitals and 18 health centers) were included using simple random sampling technique. Addis Ababa, administratively divided in to 10 sub-cities and 116 districts and the population was estimated to be 3,273,000 in 2014/15, of which 1,551,000 (47.4%) were males and 1,722,000 (52.6%) were females [37]. At the time of this study, there were a total of 90 health centers and 13 public hospitals were found in Addis Ababa. In these healthcare facilities around 7,642 healthcare providers were working.

### Study participants

All healthcare providers found in public healthcare facilities in Addis Ababa were the source population. And the study populations were randomly selected healthcare providers found in selected healthcare facilities.

### Selection criteria

All healthcare providers who were working in a selected healthcare facility who have the qualification of physicians, health officers, nurses, midwives, and anesthesiologist and work at least 6 months in the direct care of patients in public hospitals and health centers were included. Healthcare providers who were on annual and maternity leave during the data collection period were excluded.

### Sample size determination and procedure

The sample size was determined using a single population proportion formula, by considering the proportion of stethoscope disinfection after every use 50% (since there was no previous study in the study area). The following assumptions were used; 95% confidence interval (CI), 5% of marginal error, a design effect 1.5 and 10% for non-responders. Accordingly, a total of 576 healthcare providers were included.

A multi-stage sampling procedure was employed to select study participants. First, all healthcare facilities were stratified by type into hospitals and health centers. Then, twenty-one healthcare facilities were selected using a lottery method (3 hospitals and 18 health centers selected from each stratum). Afterward, the calculated sample sizes were allocated proportionally to size for each healthcare facility. Finally, systematic random sampling was employed to identify the study population from the sampling frame. And the first participant was selected randomly.

### Variables of the study and measurements

The outcome variable for this study was the healthcare provider’s stethoscope disinfection practice after every use. Stethoscope disinfection practice was assessed by asking the respondent "Do you disinfection your stethoscope?” The list of disinfection options includes “yes, after every use”, “yes, once a week or less often”, “yes, once or twice a day" and “never”. Finally, the healthcare provider’s stethoscope disinfection practices were recorded into a binary outcome. A score of “1” was assigned for acceptable disinfection practice (after every use = yes) and “0” for all other practices (no) [18].

The dependent variable studied was stethoscope disinfection after every use (yes, no). Whereas, the independent variables includes; socio-demographic characteristics (age, sex, marital status, profession, educational level, and year of service); institutional related variables (training about infection prevention, working department, and availability of standard operating procedure (SOP) in working department) and individual related variables (awareness on Ethiopian infection prevention and patient safety (IPPS) guideline, attitude towards infection prevention, knowledge towards infection prevention and control of HCAIs, self-reported infection prevention practice).

Knowledge about infection prevention and control of HCAIs was measured using the cumulative score of 17 questions each with two possible response [i.e. “yes = 1” or “no = 0”]. A scoring system was used in which the respondent’s correct and incorrect answers provided for the questions were allocated “1” or “0” points, respectively. Knowledge scores were summed up to give a total knowledge score for each healthcare provider. The total score of knowledge questions ranging from 0 to 17 was classified into two categories of response: knowledgeable (if equal to or above the mean) and not knowledgeable (below the mean). Likewise, twenty-two questions were designed to assess participants practice regarding infection prevention. To analyze the practice, similar procedures were followed a score of 1 was assigned for each acceptable or “always practice response” and 0 for unacceptable, hence the total score of infection prevention practice ranged from 0 to 22. Accordingly, participant’s infection prevention practice was classified into two categories: safe practice (if equal to and above the mean) and unsafe practice (below the mean) [27].

There were twelve questions with Likert-type scale options ranging from “strongly agree” to ‘‘strongly disagree” to assess healthcare providers attitude towards infection prevention and control of HCAIs. Accordingly, a mean value was used to classify infection prevention attitude as “favorable attitude towards infection prevention” if the score was equal or above the mean or “unfavorable attitude towards infection prevention” if the score was below the mean value [27]

### Data collection and quality control

A pre-tested interviewer-administered questionnaire was used for data collection and four trained nurse were recruited to collect the data. The data collection tool was developed by reviewing related studies [18,15] and relevant literature [20] and modified contextually. The data collection tool was first prepared in English and translated into Amharic (local language) then retranslated to English. Moreover, the questionnaire was pre-tested on 5% of the actual sample size. The completeness, consistency, and accuracy of the collected data were examined on a daily basis by two public health experts and by a principal investigator.

### Data processing and analysis

After data collection, each questionnaire was checked for completeness, missing and edited for other errors. Statistical analyses were conducted with SPSS Statistics, version 20.0 (IBM, Armonk, NY, USA). A summary descriptive statistics were computed. Bivariate and multivariable logistic regression analyses were employed to identify factors associated with disinfection practice. Variables found significant at p-value 0.05 in bivariate analysis were included in multivariable logistic regression analysis. The predicting power of variables in the final fitted model was checked by receiver observed characteristics (ROC) curve. The Hosmer and Lemeshow test as used for the overall goodness of fit. Odds ratios with 95% confidence intervals (CI) were used to determine the strength of association between the outcome and explanatory variables. The statistical significance tests were declared at the p-value < 0.05 (S1 Table).

### Ethical statement

The study was ethically approved by Addis Ababa City Administration Health Bureau Institutional Ethical Review Board (IRB). Informed written consent was obtained from each healthcare provider after explaining the purpose of the study. The right of participants to anonymity and confidentiality was maintained.

### Operational definition

Stethoscope disinfection: For the purpose of this study, stethoscope disinfection was defined as disinfecting the entire surface of the stethoscope diaphragm after every use.

## Results

### Socio-demographic characteristics

Five hundred forty-six participants took part in this study, for a response rate of 94.7%. Of these, 191 were male and 355 were female. Two hundred thirty-seven, (43.4%) were in the age group between 26 and 30 years old. The mean (standard deviation [SD]) age of the respondents was 29.19 (SD ± 6.3) and majorities 60.6% of them were married. A higher proportion (61.9%) of the respondents was first degree and above and 68.1% of healthcare providers were nurses by profession (Table 1).

**Table 1:** Socio-demographic and other characteristics of the healthcare providers in healthcare facilities in Addis Ababa, Ethiopia 2016 (N = 546).

Variable	Characteristics	Number (n)	Percentage (%)
**Age (year)**	<25	164	30.0
	25–30	237	43.4
	31–35	75	13.7
	36–40	28	5.1
	>40	42	7.7
**Sex**	Male	191	35.0
	Female	355	65.0
**Marital status**	Single	331	60.6
	Married	215	39.4
**Profession**	Nurses	372	68.1
	Health Officer	65	11.9
	Midwives	51	9.3
	Physicians*	47	8.6
	Anesthesiologist	11	2.0
**Educational status**	First degree and above	338	61.9
	Diploma	208	38.1
**Working department**	OPD, Emergency-OPD, and Triage	155	28.4
	Maternity, Delivery Gynecology and Obstetrics unit	103	18.9
	Medical and Surgical Ward	84	15.4
	Referral clinics	42	7.7
	Pediatrics ward & NICU	36	6.6
	Family planning & MCH	55	10.1
	VCT, ART & TB-clinic	42	7.6
	OR and Minor-OR	29	5.3
**Service year**	≤ 5	432	79.1
	> 5	114	20.9
**Infection prevention training**	Yes	217	39.7
	No	329	60.3
**Availability of SOP**	Yes	255	46.7
	No	291	53.3

OPD = Outpatient department, OR = Operating Room, SOP = standard operating procedure, NICU = Neonatal intensive care unit, VCT = voluntary counseling, and testing service, TB = Tuberculosis

* Resident and General Practitioner

### Characteristics of institutional and individual conditions

In this study, 217 (39.7%) of the healthcare providers were trained on infection prevention. One hundred fifty-five, (28.4%) of participates were working at the outpatient department (OPD), emergency-OPD and triage at the time of this survey. Two hundred and fifty-five (46.7%) of healthcare providers reported that they had SOP targeted on infection prevention in there working department (Table 1).

### Stethoscope disinfection practice and other individual related characteristics

In this study, 217(39.7%) [95% confidence interval [CI]:35.9, 44.0%] of healthcare providers reported that they disinfect their stethoscope after every use. Whereas majority (60.3%)[95%CI: 56.2, 64.7%] of the respondent do not disinfect their stethoscope after every use, of these 189 (34.6%)[95%CI:30.8,38.5%] of respondents never disinfect their stethoscopes, 84(15.4%)[95%CI:12.3,18.7%] disinfect once a week and 56(10.3%)[95%CI: 7.7,12.8%] of healthcare providers reported they disinfect once or twice a day (Table 2).

**Table 2:** Self-reported stethoscope disinfection practices of healthcare providers in Addis Ababa, Ethiopia, 2016 (N = 546).

Stethoscope disinfection frequency		n (%)	95% CI
Disinfect after every use ♣	Yes	217(39.7)	35.3–43.8
	No	329(60.3)	56.2–64.7
Not disinfect after every use (n = 329) ⋇	Once a week or less often	84(15.4)	12.5–18.5
	Once or twice a day	56(10.3)	7.7–12.8
	Never	189(34.6)	30.6–38.8

^**♣**^ Implies frequent disinfection

^⋇^Implies less frequent disinfection

Four hundred and fifteen (76.0%) of healthcare providers believe that stethoscope contamination can contribute to the transmission of infections. In addition, healthcare providers infection prevention (mean (SD), median) composite scores were (for knowledge = 12.43(±2.69), 13); (for practice = 13.99(± 3.33), 14) and (for attitude = 11.34(1.21), 12). Accordingly, 399(73.1%) [95%CI: 69.4, 76.7%] of healthcare providers were knowledgeable towards infection prevention and control of HCAIs and 372(68.1%) [95%CI: 64.3, 72.0] have safe infection prevention practice (S2 Table).

### Factors associated with stethoscope disinfection after every use

Tables 3–5 display the results of bivariate and multivariate logistic regression analyses to identify factors of stethoscope disinfection after every use. In bivariate analyses; age, profession, working department, infection prevention training, awareness on infection prevention and patient safety (IPPS) guidelines, availability of standard operating procedures (SOP), belief that stethoscopes can transmit HCAIs, attitude towards IP, knowledge on IP and control, and self-reported IP practice were significantly associated with stethoscope disinfection after every use. The final model was checked by Hosmer and Lemeshow test for the overall goodness of fit (0.714). In multivariate analyses, the odds of disinfection after every use was likely to be decreased by 79% among physicians compared to nurses (Adjusted odds ratio [AOR] = 0.21; 95% confidence interval [CI]: 0.09, 0.49). The odd of disinfection after every use was 1.93 times higher in healthcare providers who have awareness on infection prevention guideline than healthcare providers who did not have awareness (AOR = 1.93; 95%CI: 1.31, 2.82). Among healthcare providers, the odds of disinfection after every use were significantly higher among who had a favorable attitude towards infection prevention (AOR = 1.73, 95%CI: 1.02, 2.93), and among those have safe infection prevention practice (AOR = 3.79, 95%CI: 2.45–5.84) as compared with their counterparts.

**Table 3:** Socio-demographic factors bivariate analysis of factors associated with stethoscope disinfection after every use among healthcare providers in Ethiopia 2016.

Variables	Stethoscope disinfection after every use		COR (95%CI)	P-value
	Yes (217)	No (329)		
**Age**				
<25	65	99	0.45(0.22–0.89)*	0.02
25–30	87	150	0.39(0.20–0.77)*	0.07
31–35	30	45	0.45(0.21–0.97)*	0.04
36–40	10	18	0.38(0.14–1.02)	0.05
>40	25	17	1	
**Sex**				
Male	70	121	0.82(0.57–1.18)	0.27
Female	147	208	1	
**Profession**				
Nurses	160	212	1	
Health Officer	25	40	0.83(0.48–1.42)	0.49
Midwives	19	32	0.79(0.43–1.44)	0.43
Physicians	8	39	0.27(0.12–0.58)*	p<0.01
Anesthesiologist	5	6	1.10(0.33–3.68)	0.87
**Educational level**				
First degree and above	135	203	1.02(0.72–1.45)	0.90
Diploma	82	126	1	
**Year of service**				
≤ 5	164	268	0.70(0.46–1.07)	0.09
> 5	53	61	1	

* p< 0.05 crude, COR = Crude odds Ratio; CI = Confidence Interval

**Table 4:** Individual and institutional related factors associated with stethoscope disinfection after every use.

Variables	Stethoscope disinfection after every use		COR (95%CI)	P-value
	Yes (217)	No (329)		
**Working department**				
OPD, Emergency-OPD, and Triage	57	98	1	
Referral clinics	18	24	1.29(0.65–2.58)	0.47
Medical and Surgical Ward	42	42	1.72(1.00–2.94)*	0.04
Pediatrics ward & NICU	11	25	0.76(0.35–1.65)	0.48
Maternity, Delivery Gynecology and Obstetrics unit	36	67	0.92(0.55–1.55)	0.76
OR and Minor-OR	14	15	1.61(0.72–3.56)	0.24
FP, MCH, VCT, ART & TB-clinic	39	58	1.16(0.68–1.94)	0.58
**Infection prevention training**				
Yes	101	116	1.59(1.13–2.26)*	p<0.01
No	116	213	1	
**Do you have awareness on IPPS guidelines**				
Yes	150	184	1.76(1.23–2.53)*	p<0.01
No	67	145	1	
**Availability of SOP**				
Yes	119	136	1.72(1.23–2.44)*	p<0.01
No	98	193	1	
**Stethoscope contamination can contribute to the transmission of infections**				
Yes	177	238	1.69(1.11–2.57)*	0.01
No	40	91	1	
**Attitude towards IP**				
Favorable	192	262	1.96(1.19–3.22)*	p<0.01
Un-favorable	25	67	1	
**Knowledge towards infection prevention and control of HCAIs**				
Knowledgeable	171	228	1.65(1.10–2.46)*	0.01
Not knowledgeable	46	101	1	
**Self-reported IP practice and control of HCAIs**				
Safe	181	191	3.63(2.39–5.53)*	p<0.01
Unsafe	36	138	1	

SOP = standard operating procedure, IP = infection prevention, IPPS = infection prevention, and patient safety

* p< 0.05 crude, COR = Crude odds Ratio; CI = Confidence Interval

**Table 5:** Multivariable logistic regression analysis of factors associated with stethoscope disinfection after every use α♣.

Variables	Stethoscope disinfection after every use		AOR (95%CI)	P-value
	Yes (217)	No (329)		
**Profession**				
Nurses	160	212	1	
Health Officer	25	40	1.83(0.47–1.48)	0.53
Midwives	19	32	0.70(0.37–1.33)	0.28
Physicians	8	39	0.21(0.09–0.49)**	p<0.001
Anesthesiologist	5	6	0.86(0.24–3.01)	0.81
**Do you have awareness on IPPS guidelines**				
Yes	150	184	1.93(1.31–2.82)**	p<0.001
No	67	145	1	
**Attitude towards IP**				
Favorable	192	262	1.73(1.02–2.93)**	0.04
Un-favorable	25	67	1	
**Self-reported IP practice and control of HCAIs**				
Safe	181	191	3.79(2.45–5.84)**	p<0.001
Unsafe	36	138	1	

^**α**^ Variables included in the final model includes age, profession, department, service year, infection prevention training, awareness on IPPS guideline of Ethiopia, availability of SOP, Do you belief stethoscope contamination can contribute to the transmission of infections, attitude towards IP, knowledge on IP& control, and self-reported IP practice

** p< 0.05 adjusted, AOR = Adjusted odds Ratio; CI = Confidence Interval

^**♣**^ -2 Log likelihood = 660.68; Cox & Snell R Square = 0.125; Nagelkerke R Square = 0.170, Model p = 0.00.

## Discussion

Routine disinfection of stethoscopes after every use is one of the most important challenges among healthcare providers. In many developing countries, including Ethiopia healthcare provider’s stethoscope disinfection practice scarcely addressed in the scientific literature. For this, the study assessed stethoscope disinfection practice and associated factors among healthcare providers in central Ethiopia.

In this study, three out of four (76.0%) healthcare providers believe that stethoscope contamination can contribute to the transmission of infections. And, it was found that only a minority (39.7%) of healthcare providers reported that they disinfect their stethoscope after every use. The finding was higher when compared to a study conducted in different parts of Ethiopia, in Mizan-Tepi University Teaching Hospital (southwest Ethiopia) 6.4% [36], in Jimma University Specialized Hospital (Ethiopia) 2.8% [34] and in Tikur Anbessa Specialized Hospital (Ethiopia) 8.5% [35]. This finding could be attributed to different factors, including study setting, period and study participants. In the preceding studies conducted in Jimma and Tikur Anbessa Specialized Hospitals, they included medical students in their assessment [34, 35]. In addition, in Jimma Specialized Hospital almost all (97%) healthcare workers and medical students do not follow the standard protocol set to prevent infections. And all licensed doctors (specialist, resident and general practitioner) reported they didn’t disinfect their stethoscope regularly. Hence, 98% had contaminated stethoscope diaphragms [34].Whereas in the present study, only fulltime healthcare providers were included in the current assessment. In addition, the current study did not include specialized hospitals in the sample.

On one hand, different studies conducted elsewhere also reported infrequent disinfection practice, in Nepal (6.89%) of healthcare workers disinfecting after every use [14], in USA (24%) of healthcare workers disinfect after every patient [18], in Turkey cleaning of stethoscopes with various disinfectants at certain intervals was reported 50.4% [38], and in Pakistan, a considerably lower prevalence of stethoscope cleaning was observed 37.7% [39]. Moreover, in Nigeria, 87.9% of the participants did not clean their stethoscopes after examining each patient [40]. Furthermore, recent observational studies revealed that in 13 of 115 encounters (11.3%), the healthcare provider cleaned the stethoscope with an alcohol swab after patient interaction [41]. And another study reported, stethoscopes were disinfected per Centers for Disease Control and Prevention (CDC) guidelines in less than 4% of encounters and were not disinfected at all in 82% of encounters [42]. On the other hand, much higher stethoscopes disinfection practice was reported from a study conducted in French, 82% of medical students clean their stethoscopes regularly or from time to time [43]. This high discrepancy might be attributed to a choice of the study population, as in the cases of the French survey all respondents where medical students and in the present study the study participants were full-time health care providers.

The findings of the present study suggest that a significant number of healthcare providers in the study area and elsewhere in Ethiopia rarely disinfect their stethoscope. Which is a great concern, if the stethoscope is not cleaned or disinfected consistently the possibility of contamination with pathogenic microorganisms is likely and elevate the risk of HCAIs from one patient to another [34].

The study further found that a third of healthcare providers never disinfect their stethoscope. In fact, the finding was not validated by any observational data to verify the healthcare provider’s actual practice. Nonetheless, in many cases, the rates of stethoscope hygiene are low among healthcare providers in different settings [40–42]. And contamination of stethoscope is likely if it is not disinfected regularly. In support of this, a literature search by O’Flaherty et al across several databases for relevant studies on stethoscopes showed that stethoscopes were consistently harbor bacteria and the mean rate of stethoscope contamination across 28 studies was 85.1% (range: 47‒100%) [44]. A prospective study conducted in Swiss university teaching hospital to compare the contamination level of physicians’ hands and stethoscopes provide strong evidence of the potential for stethoscope-mediated transmission of microorganisms and the need to systematically disinfect stethoscopes after each use [45].

A study by Pal et al [46], try to identify factors associated with stethoscope disinfection and reported apprehension of damaging stethoscopes and lack of knowledge regarding good disinfectant were the underlying causes that prevent cleaning of the stethoscopes. In the same way, this study identified factors associated with stethoscope disinfection. Healthcare providers who had awareness of infection prevention guideline were more likely to disinfect their stethoscope than their counterpart healthcare providers. This could be due to the fact that as the healthcare provider’s exposure to such guidelines increase, healthcare providers are frequently exposed to appropriate stethoscopes disinfection practices and become more compliant. Since guidelines such as the Healthcare Infection Control Practices Advisory Committee Guideline for Disinfection and Sterilization in Healthcare Facilities recommended appropriate cleaning of stethoscopes with 70% ethyl or isopropyl alcohol after every use [20]. In addition, the Federal Ministry of Health of Ethiopia infection prevention and patient safety guideline strongly suggest, following contact with patient’s skin stethoscopes should be disinfected with suitable disinfectant after every use [47].

In this study, it was found that healthcare providers who had a favorable attitude towards infection prevention were almost two times more likely to disinfect their stethoscope after very use than healthcare providers who had an unfavorable attitude. The current finding was in agreement with a study conducted by Gazibara et al [48], which reported a positive correlation between a higher frequency of stethoscope cleaning and stronger positive notion that a stethoscope should be cleaned.

The results of this study showed that healthcare providers who had safe infection prevention practice were four times more likely to disinfect their stethoscope as compared to those who had unsafe infection prevention practice. This can be explained by the fact that disinfection of medical equipment that comes into contact with patients is one of the core principles of infection prevention and those healthcare workers who had good compliance towards infection prevention may have better awareness and compliance towards stethoscope disinfection. Furthermore, other factors associated with stethoscope disinfection have been documented previously. A study by Wood et al [49], identified concern for damage of stethoscope, lack of time and lack of knowledge regarding best cleaner were identified as a barrier of stethoscope cleaning [49]. A study by Hyder, also reported a history of receiving information on stethoscope cleaning has been one of the strongest predictors of stethoscope hygiene [39]. Access to disinfection material and other related issues may be also another barrier for low stethoscope disinfection practice among healthcare providers [18].

The result of this study showed that physicians were less likely to disinfect their stethoscope after every use than nurses, and this is in agreement with other studies [34,49,50]. Additionally, Shiferaw et al reported 98% of studied stethoscope diaphragms were contaminated [34]. The finding of the study is also consistent with other earlier studies, which found nurses reported to have good disinfection practice than doctors [50, 51]. Moreover, studies indicated that about 97 to 100% of doctors did not follow a standard disinfection protocol [34,50,52]. In light of this finding, a study by Uneke et al described stethoscopes used by physicians were more contaminated than those used by other health workers [40]. Interestingly Horiuchi et al in there review also reported, compared to those of the physician’s dominant hand anywhere from 70 to 100% stethoscopes are contaminated after a physical examination, and only 0–11% and 0–24% of healthcare providers disinfected their stethoscope before patient contact and after the contact respectively [53].

### Limitations of the study

The study suffered several limitations. Due to the cross-sectional nature of this study design, temporal relationships cannot be established. The study did not perform any direct observation of disinfection practices to externally validate the healthcare provider’s responses and in many cases, healthcare providers overestimate their practices. Therefore, social desirability bias is likely. The other limitation of this study is that the generalization of findings limited to public healthcare facilities. It was conducted in public healthcare facilities, thus limiting its generalizability to such settings. Unfortunately, the present study data is skewed towards nursing staff, as a result, the reader required to take precautionary measures while interpreting the finding. One additional limitation of the current study was it did not collect data regarding reasons for a reported practice (e.g. why healthcare providers do not disinfect their stethoscope). In addition, issues such as “do healthcare workers share stethoscopes or do they have their own?” and “would this changed attitude?” not addressed by this study and in need of further investigation. Lastly, subsequent observational studies are required to determine actual practice and to investigate if cleaning of stethoscopes leads to a reduction in HCAIs in healthcare settings.

## Conclusions

The available information would suggest that stethoscopes and should be disinfected between patients (after every use) to reduce the number of pathogens and risk of transmission, which is recommended by guidelines and existing literature [20,44,45,47,53]. However, the present study confirmed approximately two-fifths of the studied healthcare providers only disinfect their stethoscopes after every use. And a third of healthcare providers never disinfect their stethoscope. Factors such as awareness on infection prevention guidelines, favorable attitude towards infection prevention and safe infection prevention practice and control of HCAIs were the independent predictors of stethoscopes disinfection after every use. The study also revealed that physicians were less likely to disinfect their stethoscope compared to nurses. The findings call for clear strategies that focus on the promotion of stethoscope disinfection in healthcare facilities in Ethiopia. In addition, short term and in-service educational program to enhance stethoscope disinfection practice may have a positive effect on disinfection compliance among healthcare providers. Moreover, access to infection prevention guidelines with visual reminders (such as instructive posters) in healthcare settings should be strengthening to enhanced compliance towards stethoscope disinfection.

## Supporting information

S1 File Amharic version of the survey questionnaire. (PDF)

S2 File English version of the survey questionnaire. (PDF)

S1 Table Description of socio-demographic, institutional, and individual related variables included in this analysis. (PDF)

S2 Table Knowledge, attitude and practice of healthcare providers towards infection prevention and control of healthcare-associated infection in healthcare facilities of Addis Ababa, Ethiopia. (PDF)

S3 Table The association between stethoscope disinfection and healthcare providers profession (N = 546). (PDF)
