# Supplementary material for: Stethoscope disinfection is rarely done in Ethiopia: What are the associated factors?
Source: PLoS One. 2019 Jun 27;14(6):e0208365. doi: 10.1371/journal.pone.0208365 (PMC6597050; doi:10.1371/journal.pone.0208365)
Supplement: S1 File — (PDF) [file pone.0208365.s001.pdf]

## ክፍል አንድ፡- ማህበራዊና ዲሞግራፊክ መረጃ

| ክፍልአንድ፡- ማህበራዊና ዲሞግራፊክ መረጃ | | | | |
|---|---|---|---|---|
| ተ.ቁ | መጠይቅ | ምላሽ | ኮድ | Skip pattern |
| 101 | የሚሰሩበት የጤና ተቋም ደረጃ | [ ] ሪፈራል ሆስፒታል | 1 | |
| | | [ ] ዞናል(አጠቃላይ) ሆስፒታል | 2 | |
| | | [ ] ጤና ጣቢያ | 3 | |
| 102 | በአሁኑ ጊዜ የሚሰሩበት ክፍል | [ ] OPD, E-OPD,Triage | 1 | |
| | | [ ] የተመላላሽታካሚዎችክሊኒክ (IPD) | 2 | |
| | | [ ] የሜዲካል ክፍል እና ሰርጂካል ክፍል | 3 | |
| | | [ ] የህፃናት ክፍል | 4 | |
| | | [ ] ማዋለጃ ክፍል, Maternity እና Obstetrics | 5 | |
| | | [ ] ቀዶ ጥገና ክፍል(OR) እና ማይነር (Minor-OR) | 6 | |
| | | [ ] ላብራቶሪ | 7 | |
| | | [ ] ሌላ (ይጠቀስ) | 8 | |
| 103 | ፆታ (አትጠይቅ) | [ ] ወንድ | 1 | |
| | | [ ] ሴት | 2 | |
| 104 | ሞያ | [ ] ሀኪም | 1 | |
| | | [ ] ነርስ እና አዋላጅ ነርስ | 2 | |
| | | [ ] ጤና መኮንን | 3 | |
| | | [ ] ላብራቶሪ ቴክኒሻን | 4 | |
| | | [ ] ሌላ (ይጠቀስ) | 5 | |
| 105 | የትምህርትደረጃ | [ ] ዲፕሎማ | 1 | |
| | | [ ] የመጀመሪያ ዲግሪ | 2 | |
| | | [ ] ሁለተኛ ዲግሪ እና ከዛ በላይ | 3 | |
| 106 | እድሜ | ( ) | | |
| 107 | የስራ ዘመን ከመጨረሻ ምርቃቶት በኋላ | ( ) | | |
| 108 | የጋብቻ ሁኔታ | [ ] ያላገባ | 1 | |
| | | [ ] ባለትዳር | 2 | |
| | | [ ] የተፋታ (የተለያዩ) | 3 | |
| | | [ ] መበለት | 4 | |

## ክፍል አንድ፡ 1.2 አጠቃላይ መረጃ የጤና ባለሙያዎችን ኢንፌክሽን ፕሪቨንሽን እና እስታንዳርድ ፕሪኮኤሽን

| ክፍልአንድ፡- 1.2 Infection Prevention&Standard Precautions በተመለከተ አጠቃላይ መረጃ | | | | |
|---|---|---|---|---|
| ተ.ቁ | መጠይቅ | ምላሽ | ኮድ | |
| 109 | በስራ ሂወቶ ስለ IP ወይም SP በተመለከተ ስልጠና ወስደው | [ ] አዎ | 1 | |

| | | | | |
|---|---|---|---|---|
| | ያውቃሉ? | [ ] አይ | 2 | |
| 113 | ስለኢትዮጲያ የinfection prevention and patient safety guideline መረጃ አሎት? | [ ] አዎ | 1 | |
| | | [ ] አይ | 2 | |
| 114 | በስራላይ ያለ የ IP ኮሚቴ በመስሪያ ቤቶ አለ? | [ ] አዎ | 1 | |
| | | [ ] አይ | 2 | |
| 115 | ለምንያህልታካሚዎችንበቀንየህክምናአገልግሎቶችንይሰጣሉ ? | ( ) | | |
| 116 | በስራ ላይ ያለ SOP or Guideline or poster regarding IP መመሪያዎች አሉ? | [ ] አዎ | 1 | |
| | | [ ] አይ | 2 | |

**ክፍል አንድ: 1.3:- የጤና ባለሙያዎች የእስቴቴስኮፕ የማከምን ልምምድ (STETHOSCOPE DISINFECTION PRACTICE) በተመለከተ የቀረቡ መጠይቆች**

| ተ.ቁ | መጠይቅ | ምላሽ | ከድ | |
|---|---|---|---|---|
| | **እስቴቴስኮፕ (Stethoscope) ማከምን በተመለከተ ያሉ መጠይቆች** | | | |
| 117 | እስቴቴስኮፐህን/ሽን ታክማለህ/ታክሚያለሽ (Do you disinfect your stethoscope?) | | | |
| 117.1 | አዎ ..ከተገለገክኩበት በኋላ ወዲያውኑ | [ ] አዎ | 1 | |
| | | [ ] አይ | 2 | |
| 117.2 | አዎ..በሳምንት አንድና ከዛም በታች | [ ] አዎ | 1 | |
| | | [ ] አይ | 2 | |
| 117.3 | አዎ..በቀን አንድ እና ሁለት ግዜ (One or two a day) | [ ] አዎ | 1 | |
| | | [ ] አይ | 2 | |
| 117.4 | በፍፅም አላክምም | [ ] አይ | | |
| 118 | እስቴቴስኮፕ ለተለያዩ በሽታዎች (infections) ማስተላለፊያ ሊሆን እንደሚችል ታምናለህ | [ ] አዎ | 1 | |
| | | [ ] አይ | 2 | |

**ክፍል ሁለት፡- የ Infection Prevention (IP) ክህሎትን የተመለከቱ ጥያቄዎች?**

| | መጠይቅ | ምላሽ | ከድ | Skip pattern |
|---|---|---|---|---|
| | **ከእጅ መታጠብ ጋር የተያያዘ መጠይቅ** | | | |
| 201 | ስራ ከመጀመሮ በፊትና በኋላ እጅዎን በሳሙናና በውሀ ይታጠባሉ? | [ ] ሁልጊዜ | 2 | |
| | | [ ] አንዳንድጊዜ | 1 | |
| | | [ ] በፋፁም | 0 | |
| 202 | ታካሚዎችን ከመንካትዎ (ከማከሞ) በፊትና በኋላ እጆን ይታጠባሉ? | [ ] ሁልጊዜ | 2 | |
| | | [ ] አንዳንድጊዜ | 1 | |
| | | [ ] በፋፁም | 0 | |
| 203 | ድንገተኛ የሆነ የደም የሰውነት ፈሳሽና የተበከሉ እቃዎች በነካዎት ጊዜ እጆን በአፋጣኝ ይታጠባሉ? | [ ] ሁልጊዜ | 2 | |
| | | [ ] አንዳንድጊዜ | 1 | |
| | | [ ] በፋፁም | 0 | |

| | | | | |
|---|---|---|---|---|
| 204 | ንፁህ ጓንት ከማድረጎ በፊትና ካወለቁ በኃላ እጅን ይታጠባሉ? | [ ] ሁልጊዜ | 2 | |
| | | [ ] አንዳንድጊዜ | 1 | |
| | | [ ] በፍፁም | 0 | |
| | **የግላሽ (ጓንት)አጠቃቀምን በተመለከተ** | | | |
| 205 | የተለያዩ ታካሚዎችን ከመንካትዎ በፊት ጓንት ይቀይራሉ? | [ ] ሁልጊዜ | 2 | |
| | | [ ] አንዳንድጊዜ | 1 | |
| | | [ ] በፍፁም | 0 | |
| 206 | የተለያዩ የህክምና አገልግሎቶችን ለአንድ ታካሚ በሚሰጡበት ጊዜ ጓንት ይቀይራሉ? | [ ] ሁልጊዜ | 2 | |
| | | [ ] አንዳንድጊዜ | 1 | |
| | | [ ] በፍፁም | 0 | |
| 207 | ጠንካራ ጓንት ያደርጋሉ የህክምና መገልገያ እቃዎችን በሚያፀዱበት በሚያጥቡበት እንዲሁም በሚያክሙበት ጊዜ? | [ ] ሁልጊዜ | 2 | |
| | | [ ] አንዳንድጊዜ | 1 | |
| | | [ ] በፍፁም | 0 | |
| | **የማስክ አጠቃቀምን በተመለከተ** | | | |
| 208 | የፊት መከላከያ አልያም መሸፈኛ ያደርጋሉ የመፈንጠር (የመረጨት) አደጋ የሚያስከትልን ስራ በሚያከናውኑበት ጊዜ? | [ ] ሁልጊዜ | 2 | |
| | | [ ] አንዳንድጊዜ | 1 | |
| | | [ ] በፍፁም | 0 | |
| 209 | የ TB ተጠቂ ወይም በሽታ የያዛቸው ሰዎች በሚቀርቡበት ጊዜ የአፍና የአፍንጫ መሸፈኛ ያደርጋሉ? | [ ] ሁልጊዜ | 2 | |
| | | [ ] አንዳንድጊዜ | 1 | |
| | | [ ] በፍፁም | 0 | |
| | **የጋዎን አጠቃቀምን በተመለከተ** | | | |
| 210 | የመረጨት (የመፈንጠር) አደጋ በልብስና በአካሌ ላይ ሊያስከትል የሚችል ስራ በሚያከናውኑበት ጊዜ ሸርጥ ወይም ገዋን ይለብሳሉ? | [ ] ሁልጊዜ | 2 | |
| | | [ ] አንዳንድጊዜ | 1 | |
| | | [ ] በፍፁም | 0 | |
| | **ጎጉል አጠቃቀምን በተመለከተ** | | | |
| 211 | መከላከያ መነፅር ያደርጋሉ በፊትን እና በአፍጫዎ ላይ የመረጨተት አደጋ የሚየሰከትል ስራ በሚያከናውኑበት ጊዜ? | [ ] ሁልጊዜ | 2 | |
| | | [ ] አንዳንድጊዜ | 1 | |
| | | [ ] በፍፁም | 0 | |
| | **ፒኤፒ በተመለከተ** | | | |
| 212 | ድንገተኛየመርፌመወጋትአደጋአልያምአደገኛየሰውነትፈሳሽበ አይንወይምበአፎትላይከተረጨበኋላ PEP ለመጀመርአስበውያውቃሉ? | [ ] ሁልጊዜ | 2 | |
| | | [ ] አንዳንድጊዜ | 1 | |
| | | [ ] በፍፁም | 0 | |
| 213 | መርፌንከመክደንከማጣመምእናከመስበርይቆጠባሉ? | [ ] ሁልጊዜ | 2 | |
| | | [ ] አንዳንድጊዜ | 1 | |
| | | [ ] በፍፁም | 0 | |
| | **አደገኛ የህክምና ነክ ቆሻሻዎችን አያያዝና አወጋገድ በተመለከተ** | | | |
| 214 | መርፌንከተጠቀሙበኋላወዲያውኑበተገቢውሴፍቲቦክስውስ ጥይጥላሉ? | [ ] ሁልጊዜ | 2 | |
| | | [ ] አንዳንድጊዜ | 1 | |
| | | [ ] በፍፁም | 0 | |
| 215 | የመርፌመጣያሴፍቲቦክስለመጣልአመቺበሆነርቀትያስቀም ጣሉ? | [ ] ሁልጊዜ | 2 | |
| | | [ ] አንዳንድጊዜ | 1 | |
| | | [ ] በፍፁም | 0 | |
| 216 | ከህክምናአገልግሎትየሚወጡትንቆሻሻዎችበተገቢውመንገድ በመነጣጠልይጥላሉ? | [ ] ሁልጊዜ | 2 | |
| | | [ ] አንዳንድጊዜ | 1 | |

| | | | | |
|---|---|---|---|---|
| | | [ ] በፍፁም | 0 | |
| | **ዳግመኛ በስራ ላይ የሚውሉ የህክምና መገልገያ እቃዎችን ማከም በተመለከተ** | | | |
| 217 | የሰርጂካልመገልገያእቃዎችንከተጠቀሙበሁዋላወዲያውኑበ ማከሚያውህድውስጥይጨምራሉ? | [ ] ሁልጊዜ | 2 | |
| | | [ ] አንዳንድጊዜ | 1 | |
| | | [ ] በፍፁም | 0 | |
| 218 | ሁሉንምየግልመከላከያ (PPE) ይለብሳሉ? የተበከሉእቃዎችንበሚያጥቡናበሚያፀዱበጵጊዜ | [ ] ሁልጊዜ | 2 | |
| | | [ ] አንዳንድጊዜ | 1 | |
| | | [ ] በፍፁም | 0 | |
| 219 | የተበከሉእቃዎችንከማፅዳቶበፊትበማከሚያውህድውስጥይ ጨምራሉ? | [ ] ሁልጊዜ | 2 | |
| | | [ ] አንዳንድጊዜ | 1 | |
| | | [ ] በፍፁም | 0 | |
| | **የቲቢ በሽታን በተመለከተ** | | | |
| 220 | የሚስሉና እና የቲቢ በሽታ ተጠቂ ሊሆኑ ይችላሉ የምትላቸውን ሰዎች አፍና አፍንጫቸውን እንዲሸፍን ትነግራለህ/ሽ | [ ] ሁልጊዜ | 2 | |
| | | [ ] አንዳንድጊዜ | 1 | |
| | | [ ] በፍፁም | 0 | |
| | | [ ] በፍፁም | 0 | |
| 221 | N-95 የአፍንጫመሸፈኛማሳክበማድረግበ TB የተጠቁሰዎችንያክማሉ? | [ ] ሁልጊዜ | 2 | |
| | | [ ] አንዳንድጊዜ | 1 | |
| | | [ ] በፍፁም | 0 | |
| | **የመርፌ አሰጣጥን በተመለከተ** | | | |
| 222 | መርፌለታካሚከመስጠቶትበፊትትክክለኛውንመድሀኒትትክ ክለኛውንመጠንበትክክለኛጊዜበትክከለኛመንገድናበትክክለኛ መንገድመስጠቶንአስቀድመውያረጋግጣሉ? | [ ] ሁልጊዜ | 2 | |
| | | [ ] አንዳንድጊዜ | 1 | |
| | | [ ] በፍፁም | 0 | |
| 223 | ከጀርም ነፃ የሆነ መርፌን ይጠቀማሉ? መድሀኒት ለታካሚዎች በሚሰጡበት ጊዜ | [ ] ሁልጊዜ | 2 | |
| | | [ ] አንዳንድጊዜ | 1 | |
| | | [ ] በፍፁም | 0 | |
| | **የ IP መርሆችን በተመለከተ** | | | |
| 224 | የ IP & SP መርሆችንበስራዎትላይይታገብራሉ? | [ ] ሁልጊዜ | 2 | |
| | | [ ] አንዳንድጊዜ | 1 | |
| | | [ ] በፍፁም | 0 | |

**ክፍል ሦስት፡- የጤና ባለሙያዎችን IP አመለካከት የተመለከተ መጠይቆች (ከቀረቡት ባለ 5 ነጥብ ምላሾች ውስጥ የእርሶን አመለካከት በትክክል የሚያንጸባርቀውን አንድ ምርጫ ይምረጡ)**

| | | | | |
|---|---|---|---|---|
| 301 | በጤና ተቋማት ውስጥ የባክቴሪያ መዛመትና መሰራጨት በብዛት የሚከሰተው በጤና ባለሙያዎች የተበከለ እጅ ነው | [ ] እጥብቄእስማማለሁ | 5 | |
| | | [ ] እስማማለሁ | 4 | |
| | | [ ] አላውቅም | 3 | |
| | | [ ] አልስማማም | 2 | |
| | | [ ] እጥብቄአልስማማም | 1 | |
| 302 | የግል መከላከያ (PPE) ስራዬን ፈታኝና አታካች አያደርጉብኝም | [ ] እጥብቄእስማማለሁ | 5 | |
| | | [ ] እስማማለሁ | 4 | |
| | | [ ] አላውቅም | 3 | |
| | | [ ] አልስማማም | 2 | |

| | | | | |
|---|---|---|---|---|
| | | [ ] እጥብቄአልሰማማም | 1 | |
| 303 | እጅ መታጠብ ዋነኛው (ግምባቀደም) የ IP መከላከያ መንገድ ነው። | [ ] እጥብቄእሰማማለሁ | 5 | |
| | | [ ] እሰማማለሁ | 4 | |
| | | [ ] አላውቅም | 3 | |
| | | [ ] አልሰማማም | 2 | |
| | | [ ] እጥብቄአልሰማማም | 1 | |
| 304 | ተገቢ ያልሆነ (ደካማ) የማከም ስራ (disinfection) በጤና ባለሞያዎችና በታካሚዎች ላይ የሚከሰተውን ኢንፌክሽን (HCA) በከፍተኛ ሁኔታ ሊጨምር ይችላል። | [ ] እጥብቄእሰማማለሁ | 5 | |
| | | [ ] እሰማማለሁ | 4 | |
| | | [ ] አላውቅም | 3 | |
| | | [ ] አልሰማማም | 2 | |
| | | [ ] እጥብቄአልሰማማም | 1 | |
| 305 | ማንኛውም ሰው የጤና ባለሞያና ታካሚን ጨምሮ ለበሽታ መተላለፊያ መንገድ ሊሆኑ ይችላሉ ብሎ ማሰብየ IP መሰረታዊ መርሆ ነው። | [ ] እጥብቄእሰማማለሁ | 5 | |
| | | [ ] እሰማማለሁ | 4 | |
| | | [ ] አላውቅም | 3 | |
| | | [ ] አልሰማማም | 2 | |
| | | [ ] እጥብቄአልሰማማም | 1 | |
| 306 | መርፌን መክደን አስፈላጊ ከሆነ መከደን ያለበት በአንድ እጅ ብቻ ነው | [ ] እጥብቄእሰማማለሁ | 5 | |
| | | [ ] እሰማማለሁ | 4 | |
| | | [ ] አላውቅም | 3 | |
| | | [ ] አልሰማማም | 2 | |
| | | [ ] እጥብቄአልሰማማም | 1 | |
| 307 | በመተላለፍ ደረጃ ውስጥ ያለ ቲቢ ከታካሚው ጋር አየርን መለዋወጥ ለቲቢ በሽታ ሊያጋልጥ ይችላል። | [ ] እጥብቄእሰማማለሁ | 5 | |
| | | [ ] እሰማማለሁ | 4 | |
| | | [ ] አላውቅም | 3 | |
| | | [ ] አልሰማማም | 2 | |
| | | [ ] እጥብቄአልሰማማም | 1 | |
| 308 | ጓንት ሙሉ በሙሉ አስተማማኝ ጥበቃ ለእጃችን ስለማያደርግ ጓንትን ካወለቁ በኃላ እጅን መታጠብ እጅግ አስፈላጊ ነው፡: | [ ] እጥብቄእሰማማለሁ | 5 | |
| | | [ ] እሰማማለሁ | 4 | |
| | | [ ] አላውቅም | 3 | |
| | | [ ] አልሰማማም | 2 | |
| | | [ ] እጥብቄአልሰማማም | 1 | |
| 309 | ተገቢ የሆነ የ IP መርሆችን በጤና ተቋማት ካልተተገበሩ የጤና ተቋማት የተላላፊ በሽታና ለወረርሽን ስርጭት መንስኤ ይሆናሉ | [ ] እጥብቄእሰማማለሁ | 5 | |
| | | [ ] እሰማማለሁ | 4 | |
| | | [ ] አላውቅም | 3 | |
| | | [ ] አልሰማማም | 2 | |
| | | [ ] እጥብቄአልሰማማም | 1 | |
| 310 | ከጤና ተቋማት የሚወጡትን ቆሻሻዎች መለያየት የፅዳት ሰራተኞች የስራ ድርሻ አይደለም | [ ] እጥብቄእሰማማለሁ | 5 | |
| | | [ ] እሰማማለሁ | 4 | |
| | | [ ] አላውቅም | 3 | |
| | | [ ] አልሰማማም | 2 | |
| | | [ ] እጥብቄአልሰማማም | 1 | |
| 311 | ለ (critical) ሰርጂካል እና ሜዲካላል እቃዎችን ከጀርም ነፃ ለማድረግ ስትራላይዜሽን ዘዴ ከዲስኢንፌክሽን ዘዴ የተሻለ ተመራጭ ነው | [ ] እጥብቄእሰማማለሁ | 5 | |
| | | [ ] እሰማማለሁ | 4 | |
| | | [ ] አላውቅም | 3 | |

| | | | | |
|---|---|---|---|---|
| | | [ ] አልሰማማም | 2 | |
| | | [ ] እጥብቄአልሰማማም | 1 | |
| 312 | ለጀርሞችመተላለፊያመንገድሊሆንይችላል። Non-critical ሰርጂካል እና ሜዲካላል እቃዎችን በሚገባ ካልታከሙ ለበሽታ መተላለፍ (hospital acquired infection) ትልቅ ድርሻ ሊጫወቱ ይችላሉ። | [ ] እጥብቄእሰማማለሁ | 5 | |
| | | [ ] እሰማማለሁ | 4 | |
| | | [ ] አላውቅም | 3 | |
| | | [ ] አልሰማማም | 2 | |
| | | [ ] እጥብቄአልሰማማም | 1 | |

**ክፍል አራት፡- የጤና ባለሞያዎችን የ IP እውቀት የተመለከቱ መጠይቆች**

| ተ.ቁ | መጠይቅ | ምላሽ | ኮድ | |
|---|---|---|---|---|
| 401 | የጤና ተቋማት ውስጥ ስለሚከሰቱ በሽታዎች (*health care associated infections)* ያውቃሉ? | [ ] አዎ | 1 | |
| | | [ ] አይ | 2 | Skip to 407 |
| 402 | በጤና ተቋማት ውስጥ የሚከሰት በሽታ (HCAI) ማለት አንድ ሰው ለመታከም ከመጣበት በሽታ ተጨማሪ (ሌላ) በሽታከ48 ሰአት ቆይታ በኃላ ሲያጋጥመው የሚከሰት በሽታን ያመለክታል | [ ] አዎ | 1 | |
| | | [ ] አይ | 2 | |
| 403 | በጤና ተቋማት ውስጥ የሚከሰቱ ማናኛውም በሽታዎች (HCAI) በቀላሉ እና ብዙ ወጪ በሚያስወጣ መንገድ መከላከል ይቻላል | [ ] አዎ | 1 | |
| | | [ ] አይ | 2 | |
| | | | | |
| 404 | በጤና ተቋማት ውስጥ የሚከሰት በሽታ (HCAI) እንዴት ይተላለፋል? (ከአንድ በላይ መምረጥ ይችላሉ) | [ ] በደምና ከሰውነት በሚወጣ ፈሣሽ ንክኪ | 1 | |
| | | [ ] በተበከለ መርፌና ስለታም እቃዎች | 2 | |
| | | [ ] በአየር /በትንፋሽ/ | 3 | |
| | | [ ] በተበከለእጅ | 4 | |
| 405 | በጤና ተቋማት ውስጥ ከሚከሰቱ በሽታዎች (HCAI) በብዛት የሚከሰቱት የትኞቹ ናቸው? (ከአንድ በላይ መምረጥ ይቻላል) | [ ] ከቀዶ ጥገና በኋላ የሚከሰት እንፌክሽን (Surgical sit infection) | 1 | |
| | | [ ] የሽንት ቧንቧ ኢንፌክሽን (Urinary tract infections) | 2 | |
| | | [ ] የላይኛው የመተንፈሻ አካላት ኢንፌክሽን (Respiratory infections) | 3 | |
| | | [ ] በደም ቧንቧዎች እና በደም ላይ የሚከሰቱ ኢንፌክሽኖች (Infections of blood stream) | 4 | |
| 406 | በጤና ተቋማት ውስጥ የሚከሰቱ በሽታዎችን (HCAI) እንዴት እንከላከላለን (ከአንድ በላይ መምረጥ ይቻላል) | [ ] እጅንመታጠብ | 1 | |
| | | [ ] ቆሻሻዎችንበአግባቡማስወገድ (Proper HCWM) | 2 | |
| | | [ ] የህክምና መገልገያ እቃዎች በተገቢው ማፅዳት | 3 | |

| | | | | |
|---|---|---|---|---|
| | | [ ] ጥንቃቄ የተሞላበት መርፌ መስጠት | 4 | |
| | | [ ] የግል መከላከያ ዘዴዎችን (PPE) ባግባቡ መጠቀም | 5 | |
| | | [ ] አደገኛ በሆነ ተላላፊ በሽታ የተጠቁ ሰዎችለ ብቻ ማስተኛት | 6 | |
| 407 | የ IP ዋና መርሆች የሆኑት የትኞቹ ናቸው? (ከአንድ በላይ መምረጥ ይቻላል) | [ ] የህክምና መገልገያ እቃዎችን ማጽዳትና ማከም | 1 | |
| | | [ ] ከጤና ተቋማት የሚወጡትን አደገኛ ቆሻሳችን በአግባቡ ማስወገድ | 2 | |
| | | [ ] የግል መከላከያ ዘዴዎችን (PPE) መጠቀም | 3 | |
| | | [ ] ደህንነቱ የተጠበቀ አስተማማኝ የቀዶ ጥገና አገልግሎት መስጠት እና ደህንነቱን በጠበቀ ሁኔታ መድሀኒቶችን ለታካሚዎች መስጠት (Safe surgical procedures and medication safety) | 4 | |
| | | [ ] እጅን መታጠብ | 5 | |
| | | [ ] አስተማማኝነቱ የተጠበቀ መርፌ ለታካሚዎች መስጠት | 6 | |
| | | [ ] ከአደጋ በሁዋላ የሚወሰዱ የድንገተኛ መድሀኒቶች አቅርቦ ትአገልሎት (PPE) | 7 | |
| 408 | IP ስራዎትን ከባድ ያደርገዋል (አስቸጋሪ ያደርገዋል) | [ ] አዎ | 1 | |
| | | [ ] አይ | 2 | |

| | | | | |
|---|---|---|---|---|
| | **የእጅ ንፅህና መጠይቅ** | | | |
| 409 | እጅን መታጠብ ዋነኛውና ቀዳሚው የIP መርሆ ነው። | [ ] አዎ | 1 | |
| | | [ ] አይ | 2 | |
| 410 | **እጅን መታጠብ (Hand washing) የተመለከቱ መጠይቅ** | | | |
| | 410.1 እጅን መታጠብ ጓንት ከማድረግ በፊትና ጓንት ከማድረግ በኋላ ሊተገበር ይገባል | [ ] አዎ | 1 | |
| | | [ ] አይ | 2 | |
| | 410.2 ማንኛውንም በሽተኛ ከመንካቶ በፊትና በሁዋላ እጅን በሳሙናና በውሀ መታጠብ አስፈላጊ ነው። | [ ] አዎ | 1 | |
| | | [ ] አይ | 2 | |
| 411 | **አንድሴብቲክ የተመለከቱ መጠይቅ** | | | |
| | 411.1 ከፍተኛ ተጋላጭነት ያላቸውን ታካሚዎች ከማከማችን በፊት እጃችንን በአንድሴብቲክ (hand antisepsis)ሳሙና መታጠብ ይኖርብናል። | [ ] አዎ | 1 | |
| | | [ ] አይ | 2 | |
| | 411.2 በአልኮል የተዘጋጀ አንቲሴብቲክ( | [ ] አዎ | 1 | |

| | | | | |
|---|---|---|---|---|
| | antiseptic hand rub) ከተጠቀምን በሁዋላ እጃችን መታጠብ አይኖርብንም | [ ] አይ | 2 | |
| | 411.3 እጃችን እስካልቆሸሸ ጊዜ ድረስ አንቲ ሴብቲክን መጠቀም እጅን በውሀና በሳሙና ከመታጠብ ጋር እኩል አስተዋጽዎ አለው። | [ ] አዎ | 1 | |
| | | [ ] አይ | 2 | |
| 412 | የእጅ መታጠቢያ ሲንክ በስራዎ አቅራቢያ ይገኛል? | [ ] አዎ | 1 | |
| | | [ ] አይ | 2 | |
| 413 | በስራ ቦታዎ የማይቋረጥ የውሀ ዘቅርቦት አለ? | [ ] አዎ | 1 | |
| | | [ ] አይ | 2 | |
| 414 | **የግል መከላደከያ እቃዎች (PPE) የተመለከቱ መጠይቅ** | | | |
| | 414.1 መርፌ ለታካሚ በምንአጥበት ጊዜ ጓንት ማድረግ አያስፈልገንም | [ ] አዎ | 1 | |
| | | [ ] አይ | 2 | |
| | 414.2 ድንገተኛ የሆነ የመፈንጠር ወይም የመረጨት አደጋ ሊያሰከትል የሚችል ስራ በምንከናውንበት ጊዜ ሽርጥን መሰብሰብ አስፈላጊ ነው፡ | [ ] አዎ | 1 | |
| | | [ ] አይ | 2 | |
| | 414.3 የመረጨት አደጋ በአይናችን ላይ ሊያስከትል የሚችል ስራ በምንሰራበት ጊዜ የአይን መነፀር ማድረግ ተገቢ ነው። | [ ] አዎ | 1 | |
| | | [ ] አይ | 2 | |
| | 414.4 ሰርጂካል የአፍንጫ መሸፈኛ የTB በሽታን አይከላከልም | [ ] አዎ | 1 | |
| | | [ ] አይ | 2 | |
| 415 | ከጤና ተቋምሞት በሚሰጦት የግል መከላደከያ እቃዎች (PPE) አቅርቦት ደስተኛ ኖት? | [ ] አዎ | 1 | |
| | | [ ] አይ | 2 | |
| | **Post Exposure Prophylaxis (PEP) የተመለከቱ መጠይቅ** | | | |
| 416 | **የጤና ባለሞያው Post -exposure prophylaxis (PEP) እውቀት የተመለከቱ መጠይቅ** | | | |
| | 425.1 PEP ከሁለት ሰአት በፊት መውሰድ የተከሰተውን አደጋ ለማስቀረት ተመራጩ ነው | [ ] አዎ | 1 | |
| | | [ ] አይ | 2 | |
| | 425.2 PEP ከ72 ሰአት በኋላ መውሰድ እምብዛም ጥቅም አይኖረውም | [ ] አዎ | 1 | |
| | | [ ] አይ | 2 | |
| | 425.3 PEP የሚሰጠው ለ28 ቀናት ነው። | [ ] አዎ | 1 | |
| | | [ ] አይ | 2 | |
| | **TB infection control የተመለከቱ መጠይቅ** | | | |
| 417 | **TB-IC እውቀት የተመለከቱ መጠይቅ** | | | |
| | 417.1 በ BCG የተከተቡ ሰዎች በ TB ፈፅሞ ሊጠቁ አይችሉም | [ ] አዎ | 1 | |
| | | [ ] አይ | 2 | |
| | 417.2 በጤና ተቋም ውስጥ የሚያስሉና የሚያስነጥሱ ታካሚዎችን ባፋጣኝ እርዳታ በመስጠት በጤና ተቋማት ውስጥ የሚዎዩበትን ጊዜ ማሳጠር የጤና ባለሙያዎች ሀላፊነት ነው። | [ ] አዎ | 1 | |
| | | [ ] አይ | 2 | |
| | **ሴፍ ኢንጄክሽን (Safe injection) በተመለከተ** | | | |
| 418 | **የ Safe injection እውቀት የተመለከቱ መጠይቅ** | | | |
| | 418.1 ሴፍኢንጄክሽን ማለት ስንል ታካሚውንና የጤና ባለሞያውን ከአደጋ የጠበቀ እና የማይጎዳ ብሎም አግባብነት ያለውን የአወጋገድ ስርአት | [ ] አዎ | 1 | |
| | | [ ] አይ | 2 | |

| | | | | |
|---|---|---|---|---|
| | የተከተለ ማለታችን ነው፡፡ | | | |
| | 418.2 መርፌ ከመስጠት በፊት በአንቶሴኮቲክ ቆዳን መጥረግ አስፈላጊ ነው፡፡ | [ ] አዎ | 1 | |
| | | [ ] አይ | 2 | |
| | 418.3 የተጠቀምንባቸው መርፌዎች በሴፍቲቦክስ ብቻ መጣል ይኖርብናል | [ ] አዎ | 1 | |
| | | [ ] አይ | 2 | |
| | 418.4 HBV & HCV ጥንቃቄ በጎደለው መርፌ ሊተላለፍ ይችላል፡፡ | [ ] አዎ | 1 | |
| | | [ ] አይ | 2 | |
| | **የህክምና መገልገያ እቃዎችን ማከም (Instrument Processing )የተመለከቱ መጠይቅ** | | | |
| 419 | ስለ Spaulding ክፍፍል ሰምተው ያውቃሉ? | [ ] አዎ | 1 | |
| | | [ ] አይ | 2 | ወደ 421 |
| 420 | ከ spaulding ክፍል ውስጥ የሰውነት ውስጠኛውን ክፍልየሚነኩ እቃዎችእና የህክምና አገልግሎቶች ከርቲካል(Critical) በሚባለው ምድብ ወስጥይመደባሉ | [ ] አዎ | 1 | |
| | | [ ] አይ | 2 | |
| 421 | 0.5% በበረኪና ውህድን ማዘጋጀት ይችላሉ? | [ ] አዎ | 1 | |
| | | [ ] አይ | 2 | |
| | **ዲኮንታሚኔሽን (Decontamination)** | | | |
| 422 | **የ ዲኮንታሚኔሽን (Decontamination) እውቀት የተመለከቱ መጠይቅ** | | | |
| | 422.1 ዲኮንታሚኔሽን የህክምና እቃዎችን ለማከም የመጀመሪያው ቀዳሚ ተግባር ነው | [ ] አዎ | 1 | |
| | | [ ] አይ | 2 | |
| | 422.2 የ decontamination ዋና አላማ የጤና ባለሞያዎችንና አጋር ባልደረቦችን ከድንገተኛ አደጋ መከላከል ነው፡፡ | [ ] አዎ | 1 | |
| | | [ ] አይ | 2 | |
| | 422.3 decontamination አደገኛ የሆነ ጀርሞችን HIV HBV እና HCV ፍዝ (inactivate) ያደራጋል ቁጥራቸውንም ይቀንሳል፡፡ | [ ] አዎ | 1 | |
| | | [ ] አይ | 2 | |
| | 422.4 በ0.5% በረኪና ውህድ ውስጥ ለ10 ደቂቃ እቃዎችን መዘፍዘፍ Decontamination እንዲፈጠር ያደርጋል፡፡ | [ ] አዎ | 1 | |
| | | [ ] አይ | 2 | |
| | **የዲስኢንፌክሽን (Disinfection) እውቀት የተመለከቱ መጠይቅ** | | | |
| 423 | **የDisinfection እውቀት የተመለከቱ መጠይቅ** | | | |
| | 423.1 Disinfection ሁሉንም ጀርሞች የባክቴሪያ እስፐርን (spores) ጨምሮ ሁሉንም ፈፅሞ ይገላል፡፡ | [ ] አዎ | 1 | |
| | | [ ] አይ | 2 | |
| | 423.2 ከ Decontamination እና ከማፅዳት በሁዋላ የህክምና መገልገያ እቃዎችን በ0.1% የበረኪና ውህድ ውስጥ ለ 20 ደቂቃ መዘፍዘፍ high level disinfection (HLD) ይሰጠናል | [ ] አዎ | 1 | |
| | | [ ] አይ | 2 | |
| | **የእስቴራይላዜሽን (Sterilization) የተመለከቱ መጠይቅ** | | | |
| 424 | **የsterilization እውቀት የተመለከቱ መጠይቅ** | | | |
| | 424.1 steralization ሁሉን ምጀርሞች የባክቴሪያ እስፐርን (spores) ጨምሮ ሁሉንም አረቂቅ ተዋሲያን ፈፅሞ ይገላል፡፡ | [ ] አዎ | 1 | |
| | | [ ] አይ | 2 | |
| | 424.2 የሰርጂካል እቃዎችን ከጀርም ነፃ ለማድረግ | [ ] አዎ | 1 | |

| | | | | |
|---|---|---|---|---|
| | steam sterilization ተመራጭ መንገድ ነው። | [ ] አይ | 2 | |
| | 424.3 Sterilization በእንፋሎት (high-pressure steam) በደረቅ ሙቀት (dry heat) እና በኬሚካሎች(Glutaraldehyde and Formaldehyde) መተግበር እንችላለን። | [ ] አዎ | 1 | |
| | | [ ] አይ | 2 | |
| | **ከህክምና አገልግሎት የሚወጡትን ቆሻሻዎች አወጋገድን (HCWM) የተመለከቱ መጠይቅ** | | | |
| 425 | **ከህክምና አገልግሎት የሚወጡትን ቆሻሻዎች የተመለከቱ መጠይቅ** | | | |
| | 425.1 ሴፍቲቦክስ(safety box ) ¾ ሲሞላ መዘጋት እና መጣል ይኖርበታል። | [ ] አዎ | 1 | |
| | | [ ] አይ | 2 | |
| | 425.2 ከህክምና አገልግሎት የሚወጡትን ቆሻሻዎች(Health care wastes) በአግባቡ መለያየት (segregation) የአምራች(generator) ሀላፊነት ነው። | [ ] አዎ | 1 | |
| | | [ ] አይ | 2 | |
| | 425.3 ከህክምና አገልግሎት የሚወጡትን አደገኛ ቆሻሻዎች መለያያ እና መጣያ 3 ባልደዎች አሉ (for general, infectious and sharp wastes) | [ ] አዎ | 1 | |
| | | [ ] አይ | 2 | |

***"ግዜዎትን ሰውተው ለሰጡት መልስ ከልብ አመሰግናለው!!!"***
