# Supplementary material for: Stethoscope disinfection is rarely done in Ethiopia: What are the associated factors?
Source: PLoS One. 2019 Jun 27;14(6):e0208365. doi: 10.1371/journal.pone.0208365 (PMC6597050; doi:10.1371/journal.pone.0208365)
Supplement: S2 File — (PDF) [file pone.0208365.s002.pdf]

PART ONE: 1.1 SOCIO-DEMOGRAPHIC DATA *(Please, thick on the space provided)*

**1.1 SOCIO DEMOGRAPHIC DATA**				
S.No	**Item/Questions**	**Response option**	**Code**	**Skip pattern**
101	Level of Health Institution	[ ] Referral hospital	1	
		[ ] General /zonal/hospital	2	
		[ ] Health center	3	
102	Current working Department	[ ] OPD , E-OPD and Triage	1	
		[ ] Inpatient clinic	2	
		[ ] Medical and Surgical	3	
		[ ] Pediatrics	4	
		[ ] Maternity, Obstetrics and Delivery room	5	
		[ ] OR and Minor-OR	6	
		[ ] Laboratory	7	
		[ ] Other (specify)	8	
103	Sex (*do not ask*)	[ ] Male	1	
		[ ] Female	2	
104	What is your Profession?	[ ] Doctor	1	
		[ ] Nurse/Midwife	2	
		[ ] Health officer	3	
		[ ] Lab technicians	4	
		[ ] Other (specify)	5	
105	What is your Educational status?	[ ] Diploma	1	
		[ ] 1st degree	2	
		[ ] 2nd degree and above	3	
106	What is your current age?	( ) years		
107	Years of service after the last graduation?	( ) years		
108	What is your Marital stat1us?	[ ] Single	1	
		[ ] Married	2	
		[ ] Divorce/ separated	3	
		[ ] Widowed	4	

PART ONE: - 1.2 GENERAL INFORMATION ON IP AND SP

**1.2 GENERAL INFORMATION ON IP AND SP**				
S.No	Item/Questions	Response option	Code	Skip pattern
109	*Have you ever participated in any training program about infection prevention or standard precautions?*	[ ] Yes	1	
		[ ] No	2	
113	*Have you any information on IP and patient safety guideline of Ethiopia?*	[ ] Yes	1	
		[ ] No	2	
114	*IP committee in your facility?*	[ ] Yes	1	
		[ ] No	2	
115	*For how many patients do you provide service (care) per day?*	( ) number		
116	*Is there SOP, Guideline or poster that describe Infection*	[ ] Yes	1	

	prevention issues in your department?	[ ] No	2	

PART ONE: 1.3:- HCWS STETHOSCOPE DISINFECTION PRACTICE AND RELATED QUESTIONS

S.no	Item/Questions	Response option	Code	Skip pattern
	**Stethoscope disinfection characteristics**			
117	Do you disinfect your stethoscope?			
117.1	Yes “after every use?”	[ ] Yes	1	
		[ ] No	2	
117.2	Yes “Once a week or less often”	[ ] Yes	1	
		[ ] No	2	
117.3	Yes “One or two a day”	[ ] Yes	1	
		[ ] No	2	
117.4	Never	[ ] No	1	
118	Do you belief stethoscope contamination can contribute to the transmission of infections	[ ] Yes	1	
		[ ] No	2	

PART TWO: - INFECTION PREVENTION PRACTICES OF HCWS

(please select only one response by ticking in the space provided)

**S.no**	**Item/Questions**	**Response option**	**Code**	**Skip pattern**
	**Hand washing**			
201	Do you wash your hand with soap and water at the beginning of each day on arrival and after leaving the work place?	[ ] Always	2	
		[ ] Sometimes	1	
		[ ] Never	0	
202	Do you wash your hand before any direct contact and after every patient contact?	[ ] Always	2	
		[ ] Sometimes	1	
		[ ] Never	0	
203	Do you wash immediately your hand after accidental contact with blood, body fluid, secretions and contaminated item or instruments?	[ ] Always	2	
		[ ] Sometimes	1	
		[ ] Never	0	
204	Do you wash your hand before wearing and after removing sterile or examination gloves?	[ ] Always	2	
		[ ] Sometimes	1	
		[ ] Never	0	
	**Use of glove**			
205	Do you change glove between every patient contact?	[ ] Always	2	
		[ ] Sometimes	1	
		[ ] Never	0	
206	Do you change glove between different procedures on the same patient?	[ ] Always	2	
		[ ] Sometimes	1	
		[ ] Never	0	
207	Do you wear Heavy Duty Utility gloves while handling, decontaminating, cleaning or disinfecting contaminated items?	[ ] Always	2	
		[ ] Sometimes	1	
		[ ] Never	0	
	**Use of Mask**			
208	Do you wear facemask or face shield when	[ ] Always	2	

	undertaking procedures likely to generate splashes?	[ ] Sometimes	1	
		[ ] Never	0	
209	Do you wear mask that cover both nose and mouth whenever approaching to TB suspected and confirmed patients?	[ ] Always	2	
		[ ] Sometimes	1	
		[ ] Never	0	
	**Gown/apron**			
210	Do you wear gown or apron during procedures and/or patient care activities when contact of clothing or exposed skin with blood/body fluids, secretions, and excretions is anticipated?	[ ] Always	2	
		[ ] Sometimes	1	
		[ ] Never	0	
	**Goggle or Eyewear**			
211	Do you wear goggle or eye protection during all patient procedures and /or patient care activities likely to generate splashes or sprays of blood, body fluids or secretions into the eye and mouth?	[ ] Always	2	
		[ ] Sometimes	1	
		[ ] Never	0	
	**PEP and Occupational injury**			
212	Do you ever consider or believe to starting post exposure prophylaxes (PEP) after a needle stick injury or accidental infectious body fluid splashes on eye, mouth or mucous membranes?	[ ] Always	2	
		[ ] Sometimes	1	
		[ ] Never	0	
213	Do you avoid recapping, bending, breaking and hand-manipulating used needles	[ ] Always	2	
		[ ] Sometimes	1	
		[ ] Never	0	
	**HCWM**			
214	Do you dispose sharps or needles immediately after use in puncture proof container or safety box?	[ ] Always	2	
		[ ] Sometimes	1	
		[ ] Never	0	
215	Do you put sharp object disposal container as close as hand reached places?	[ ] Always	2	
		[ ] Sometimes	1	
		[ ] Never	0	
216	Do Segregation healthcare wastes before disposal in to general, infectious and sharp wastes.	[ ] Always	2	
		[ ] Sometimes	1	
		[ ] Never	0	
	**Instrument processing**			
217	Do you immediately immerse surgical instruments or critical items or any reusable items in decontaminant solution after use?	[ ] Always	2	
		[ ] Sometimes	1	
		[ ] Never	0	
218	Do you wear all PPE (mask, eyewear, apron and heavy duty utility gloves) during instrument processing?	[ ] Always	2	
		[ ] Sometimes	1	
		[ ] Never	0	
219	Do you perform decontamination before cleaning	[ ] Always	2	
		[ ] Sometimes	1	
		[ ] Never	0	
	**TB infection control and Cough hygiene**			
220	Do you Instruct symptomatic persons to cover mouth/nose when sneezing/coughing to use handkerchief and dispose in no-touch receptacle?	[ ] Always	2	
		[ ] Sometimes	1	
		[ ] Never	0	

221	Do you wear N-95 mask when treating patients with active or suspected pulmonary TB.	[ ] Always	2	
		[ ] Sometimes	1	
		[ ] Never	0	
	**Injection safety**			
222	Do you check and verify before administering injection it is given to the right person, the right drug, the right dose, the right time, the right route and the right injection equipment?	[ ] Always	2	
		[ ] Sometimes	1	
		[ ] Never	0	
223	Do you use Sterile syringes and sterile needles while administering all medication (Intramuscular, Intravenous and Intradermal) or collect blood from patients?	[ ] Always	2	
		[ ] Sometimes	1	
		[ ] Never	0	
	**Infection prevention principles**			
224	Do you follow infection prevention and standard precautions principles in your facility?	[ ] Always	2	
		[ ] Sometimes	1	
		[ ] Never	0	

PART THREE: - ATTITUDE OF HCWs TO WARD IP ***(please select only one response by ticking in the space provided best suits among the Likert-scale measurements of your attitude)***

301	Spreading of bacteria in health facilities mainly occurred via the hands of HCWs	[ ] Strongly agree	5	
		[ ] Agree	4	
		[ ] Don't know	3	
		[ ] Disagree	2	
		[ ] Strongly disagree	1	
302	PPE does not make work difficult	[ ] Strongly agree	5	
		[ ] Agree	4	
		[ ] Don't know	3	
		[ ] Disagree	2	
		[ ] Strongly disagree	1	
303	Hand hygiene is the single most important infection prevention procedure.	[ ] Strongly agree	5	
		[ ] Agree	4	
		[ ] Don't know	3	
		[ ] Disagree	2	
		[ ] Strongly disagree	1	
304	Non appropriate disinfection procedure increase the risk of transmitting of infections or HCAIs among hospitalized patients and HCWs	[ ] Strongly agree	5	
		[ ] Agree	4	
		[ ] Don't know	3	
		[ ]Disagree	2	
		[ ] Strongly disagree	1	
305	Considering Every Person (patient or staff) as a sources means and susceptible for infection transmission is one of the key principles of prevention infections.	[ ] Strongly agree	5	
		[ ] Agree	4	
		[ ] Don't know	3	
		[ ] Disagree	2	
		[ ] Strongly disagree	1	
306	If needle recapping is necessary it is only performed by using a single handed scoop	[ ] Strongly agree	5	
		[ ] Agree	4	
		[ ] Don't know	3	

	technique only	[ ] Disagree	2	
		[ ] Strongly disagree	1	
307	Anyone who share air with a person with pulmonary TB in an infectious stage is at risk	[ ] Strongly agree	5	
		[ ] Agree	4	
		[ ] Don't know	3	
		[ ] Disagree	2	
		[ ] Strongly disagree	1	
308	Glove does not provide a complete protection and hand washing is necessary after removing gloves	[ ] Strongly agree	5	
		[ ] Agree	4	
		[ ] Don't know	3	
		[ ] Disagree	2	
		[ ] Strongly disagree	1	
309	In the absence of infection prevention or standard precautions health care facilities can be the source of infection and epidemic diseases	[ ] Strongly agree	5	
		[ ] Agree	4	
		[ ] Don't know	3	
		[ ] Disagree	2	
		[ ] Strongly disagree	1	
310	Segregation health care wastes at the point of waste generation the duty of waste handlers	[ ] Strongly agree	5	
		[ ] Agree	4	
		[ ] Don't know	3	
		[ ] Disagree	2	
		[ ] Strongly disagree	1	
311	Sterilization is the preferred method of instrument processing for critical patient care items and devices.	[ ] Strongly agree	5	
		[ ] Agree	4	
		[ ] Don't know	3	
		[ ] Disagree	2	
		[ ] Strongly disagree	1	
312	Non-critical medical and surgical devices when they are not disinfected can play a contributory role in the transmission of hospital acquired infection	[ ] Strongly agree	5	
		[ ] Agree	4	
		[ ] Don't know	3	
		[ ] Disagree	2	
		[ ] Strongly disagree	1	

PART FOUR: - KNOWLEDGE OF HCWs ON INFECTION PREVENTION *(please, thick the response on the space provided)*

S.No	Item/Questions	Response option	Code	Skip pattern
	**Health Care Associated Infection(HCAI)**			
401	*Do you know the standard definition of heard of health care associated infections (HCAIs)?*	[ ] Yes	1	If NO skip to Q 407
		[ ] No	2	
402	Health-care-associated infection (HCAI) refers to infection that is acquired during the process of care and not manifested at the time of admission to a hospital.	[ ] Yes	1	
		[ ] No	2	
403	HCAIs can be prevented with readily available, relatively inexpensive strategies	[ ] Yes	1	
		[ ] No	2	
404	*How does HCAIs transmitted **(more***	*[ ] Through blood and body fluid*	1	

	than one answer is possible)	*contact*		
		[ ] Through contaminated needles and sharps	2	
		[ ] Through Air droplet	3	
		[ ] Through contaminated hands	4	
405	*The most frequent HCAIs includes*			
	(more than one answer is possible)	*[ ] Surgical sit infection*	1	
		[ ] Urinary tract infections	2	
		[ ] Respiratory infections	3	
		[ ]Infections of blood stream	4	
406	*HCAIs can be prevented by applying*			
	*(**more than one answer is possible**)*	*[ ] Hand Washing*	1	
		[ ] Proper HCWM	2	
		[ ] Instrument processing	3	
		[ ] Safe injection practices	4	
		[ ] Appropriate use of PPE	5	
		[ ] Isolation of potentially infectious person	6	
407	*What does the concept of infection prevention implies?(more than one answer is possible)*	*[ ] Instrument processing*	*1*	
		[ ] HCWM	*2*	
		[ ] use of PPE	*3*	
		[ ] Safe surgical procedure and medication safety	*4*	
		[ ] Hand hygiene	*5*	
		[ ] Safe injection practice	*6*	
		[ ] PEP	*7*	
408	*Do you think infection prevention make your work harder*	[ ] Yes	1	
		[ ] No	2	
	**Hand Hygiene**			
409	Hand hygiene is the single most critical infection prevention procedure	[ ] Yes	1	
		[ ] No	2	
410	**Knowledge on hand washing**			
	410.1 Hand washing must be performed before using and after removing sterile or examination gloves	[ ] Yes	1	
		[ ] No	2	
	410.2 Hand washing must be performed before and after coming in direct contact with patient or client	[ ] Yes	1	
		[ ] No	2	
411	**Knowledge on hand antisepsis and antiseptic hand rub**			
	411.1 Hand antisepsis be applied before examination or caring highly susceptible patients	[ ] Yes	1	
		[ ] No	2	
	411.2 After applying Antiseptic hand-rub it is not necessary to rinse hands with water	[ ] Yes	1	
		[ ] No	2	

	411.3 Alcohol-based hand sanitizer was as effective as soap and water when hands were not visibly dirty	[ ] Yes	1	
		[ ] No	2	
412	*Does your department (work places) have hand washing facility or sink ?*	[ ] Yes	1	
		[ ] No	2	
413	*Does your department have a continuous running water supply for hand washing*	[ ] Yes	1	
		[ ] No	2	
	**Personal protective Equipment's**			
414	**Knowledge on use of PPE (Glove, Apron, Goggle and Mask use)**			
	414.1 Glove should must be worn while giving injections	[ ] Yes	1	
		[ ] No	2	
	414.2 Aprons or gowns should always wear if the risk of contamination with aggressive procedures and/or activities performed.	[ ] Yes	1	
		[ ] No	2	
	414.3 Goggles should be worn when needed to avoid exposure to eyes	[ ] Yes	1	
		[ ] No	2	
	414.4 Surgical mask cannot protect the HCWs from being infected by TB	[ ] Yes	1	
		[ ] No	2	
415	*Do you have the necessary PPE in your department?*	[ ] Yes	1	
		[ ] No	2	
	**Post Exposure Prophylaxis (PEP)**			
416	**Knowledge on Post -exposure prophylaxis (PEP) recommendations**			
	416.1 PEP is preferred when initiated within 2 hours of exposure.	[ ] Yes	1	
		[ ] No	2	
	416.2 PEP is not effective when initiated after 72 hours of exposure	[ ] Yes	1	
		[ ] No	2	
	416.3 The duration of the regimens of Post -exposure prophylaxis (PEP) is 28 days	[ ] Yes	1	
		[ ] No	2	
	**TB infection control**			
417	**Knowledge on TB-IC**			
	417.1People that received BCG vaccination do not develop active TB	[ ] Yes	1	
		[ ] No	2	
	417.2 HCWs should be responsible to triage and minimize the time a TB patient spend in health facility	[ ] Yes	1	
		[ ] No	2	
	**Safe injection**			
418	**Knowledge on Safe injection**			
	418.1 Injection safety refers to injections which does not harm the recipient, and does not expose provider to any avoidable risks and does not result waste that is dangerous to the community.	[ ] Yes	1	
		[ ] No	2	

	418.2 Swabbing of clean skin with antiseptic solution prior to giving injection is unnecessary	[ ] Yes	1	
		[ ] No	2	
	418.3 Used syringes and needles should only discarded in a sharp waste box alone	[ ] Yes	1	
		[ ] No	2	
	418.4 HBV and HCV could be transmitted via unsafe injections	[ ] Yes	1	
		[ ] No	2	
	**INSTRUMENT PROCESSING**			
419	*Have you ever heard of the Spaulding categories of potential infection risks*	[ ] Yes	1	
		[ ] No	2	If no go to Q421
420	From Spaulding categories items and practices affect mucous membrane and small areas of intact skin consider as critical items	[ ] Yes	1	
		[ ] No	2	
421	Do you know how to preparing 0.5% chlorine solution?	[ ] Yes	1	
		[ ] No	2	
	**DECONTAMINATION**			
**422**	**Knowledge on decontamination**			
	422.1 Decontamination is the first step in instrument processing practices	[ ] Yes	1	
		[ ] No	2	
	422.2 The objective of decontamination is to protect HCWs and supportive staffs while handling instruments	[ ] Yes	1	
		[ ] No	2	
	422.3 Decontamination inactivate and reduce the number of HBV,HCV and HIV	[ ] Yes	1	
		[ ] No	2	
	422.4 Decontamination achieved by soaking items in 0.5% chlorine solution for 10min	[ ] Yes	1	
		[ ] No	2	
	**DISINFECTION**			
423	Knowledge on disinfection			
	423.1 Disinfection is a process that all the living microorganisms including bacterial spores are killed.	[ ] Yes	1	
		[ ] No	2	
	423.2 After decontamination and cleaning 0.1% chlorine solution for 20 min provide High Level Disinfection (HLD).	[ ] Yes	1	
		[ ] No	2	
	**STERILIZATION**			
424	Knowledge on sterilization of items			
	424.1 Sterilization is a process where all the living microorganisms, including bacterial spores are killed.	[ ] Yes	1	
		[ ] No	2	
	424.2 Steam sterilization is a preferred method for reusing surgical instruments	[ ] Yes	1	
		[ ] No	2	
	424.3 Sterilization can be achieved by high-pressure steam, dry heat, or Chemical sterilants such as Glutaraldehyde and Formaldehyde.	[ ] Yes	1	
		[ ] No	2	
	**HCWM**			
425	Knowledge on waste management			

	425.1 Safety boxes should close and sealed when it is ¾ full?	[ ] Yes	1	
		[ ] No	2	
	425.2 Waste segregation is the responsibility of waste generators	[ ] Yes	1	
		[ ] No	2	
	425.3 Is there separate(preferred if color coded) and leveled waste collection container available for general, infectious and sharp wastes	[ ] Yes	1	
		[ ] No	2	

Thank you with all the respect.
