# Supplementary material for: Stethoscope disinfection is rarely done in Ethiopia: What are the associated factors?
Source: PLoS One. 2019 Jun 27;14(6):e0208365. doi: 10.1371/journal.pone.0208365 (PMC6597050; doi:10.1371/journal.pone.0208365)
Supplement: S1 Table — (PDF) [file pone.0208365.s003.pdf]

S1 Table: Description of socio-demographic, institutional, and individual related variables included in this analysis.

Variable description	Format for analysis
Socio-demographic characteristic	
Age of healthcare providers (years)	Categorical variable, reference category was healthcare providers' age above 40 years
Sex	Binary, reference category was female sex
Profession	Categorical variable, reference category was nurses
Educational level	Binary, reference category was diploma healthcare providers
Year of service	Binary, reference category was above five years work experience
Healthcare facility related variables	
Training about infection prevention	Binary, reference category was no history of training about infection prevention
Working department	Categorical variable, reference category was OPD, Emergency-OPD and Triage
Availability of SOP in working department	Binary, reference category was no SOP available in working department
Individual related variables	
Awareness on Ethiopian IPPS guideline	Binary, reference category was no awareness regarding Ethiopian IPPS guideline

Do you think transmission of infections occurs via stethoscopes	Binary, reference category was no
Attitude towards infection prevention	Binary, reference category was unfavorable attitude towards infection prevention
Knowledge towards preventing HCAIs	Binary, reference category was not knowledgeable towards preventing HCAIs
Self-reported infection prevention practice	Binary, reference category was unsafe infection prevention practice
