# Supplementary material for: Stethoscope disinfection is rarely done in Ethiopia: What are the associated factors?
Source: PLoS One. 2019 Jun 27;14(6):e0208365. doi: 10.1371/journal.pone.0208365 (PMC6597050; doi:10.1371/journal.pone.0208365)
Supplement: S2 Table — (PDF) [file pone.0208365.s004.pdf]

S2 Table: Knowledge, attitude and practice of healthcare providers towards infection prevention and control of healthcare associated infection in healthcare facilities of Addis Ababa, Ethiopia.

**Study variable**	**N (%)**	**Infection prevention Knowledge score**		**Infection prevention attitude score**		**Infection prevention practice score**	
		**Mean score**	**± SD**	**Mean score**	**± SD**	**Mean score**	**± SD**
**Profession**							
Nurses	372 (68.1)	12.31	2.88	11.32	1.23	14.03	3.39
Health Officer	65 (11.9)	12.37	2.52	11.31	1.22	13.29	3.63
Midwives	51 (9.3)	12.80	2.24	11.33	1.21	14.45	3.08
Physicians	47 (8.6)	12.94	1.69	11.47	1.12	13.94	2.83
Anesthesiologist	11 (2.0)	12.91	2.50	11.55)	0.68	14.91	2.42
**Overall composite score**	**546 (100)**	**12.43**	**2.69**	**13.99**	**3.33**	**11.34**	**1.21**
