# Supplementary material for: Stethoscope disinfection is rarely done in Ethiopia: What are the associated factors?
Source: PLoS One. 2019 Jun 27;14(6):e0208365. doi: 10.1371/journal.pone.0208365 (PMC6597050; doi:10.1371/journal.pone.0208365)
Supplement: S3 Table — (PDF) [file pone.0208365.s005.pdf]

S3 Table: The association between self reported stethoscope disinfection practices and healthcare providers profession in Addis Ababa, Ethiopia, 2016 (N=546).

	Profession	Nurses	Health officers	Midwives	Physicians	Anesthesiologist	Total	X^2(df), p-value
Stethoscope disinfection practices	Disinfect after every use	160	25	19	8	5	217	12.11(4), 0.016
		73.7%	11.5%	8.8%	3.7%	2.3%	100.0%	
	Once a week or less often	62	13	3	4	2	84	6.84(4), 0.144
		73.8%	15.5%	3.6%	4.8%	2.4%	100.0%	
	One or two a day	36	5	4	10	1	56	7.14(4), 0.129
		64.3%	8.9%	7.1%	17.9%	1.8%	100.0%	
	Never	114	22	25	25	3	189	14.71(4), 0.005
		60.3%	11.6%	13.2%	13.2%	1.6%	100.0%	

X^2(df) = Chi-square test (degree of freedom)
